# Association Between Single Nucleotide Polymorphisms and Viral Load in Congenital Cytomegalovirus Infection

**DOI:** 10.34763/jmotherandchild.20202404.d-20-00014

**Published:** 2021-07-16

**Authors:** Dominika Jedlińska-Pijanowska, Beata Kasztelewicz, Anna Dobrzańska, Katarzyna Dzierżanowska-Fangrat, Maciej Jaworski, Justyna Czech-Kowalska

**Affiliations:** 1 Department of Neontology and Neonatal Intensive Care , The Children’s Memorial Health Institute, Warsaw, Poland; 2 Department of Clinical Microbiology and Immunology, The Children’s Memorial Health Institute, Warsaw, Poland; 3 Department of Biochemistry, Radioimmunology and Experimental Medicine, The Children’s Memorial Health Institute, Warsaw, Poland

**Keywords:** viral load, single nucleotide polymorphism, congenital cytomegalovirus infection, newborn

## Abstract

**Background:**

There are limited data on factors that determine viral load (VL) in congenital cytomegalovirus (cCMV) infection. Single nucleotide polymorphisms (SNPs) might influence individual host response to infection. This study aimed to investigate the association between SNPs in genes encoding cytokines or cytokine receptors and VL in newborns with cCMV.

**Material and methods:**

Eight polymorphisms (IL1B rs16944, IL12B rs3212227, IL28B rs12979860, CCL2 rs1024611, DC-SIGN rs735240, TLR2 rs5743708, TLR4 rs4986791 and TLR9 rs352140) were analyzed in study population of 233 newborns, including 92 cCMV-infected newborns (73 symptomatic and 19 asymptomatic) by TaqMan SNP Predesigned Genotyping Assays. The association analysis was performed using SNPStats software and STATISTICA10.

**Results:**

The association between IL12B polymorphism and viruria was observed (*p* = 0.029). In multiple comparison tests, heterozygous T/G genotype of IL12B was associated with higher viruria than T/T genotype (*p* = 0.041) in cCMV-infected newborns. In allele analysis, T allele of IL12B was associated with higher viremia (*p* = 0.037) in symptomatic newborns. We observed higher VL in symptomatic newborns in comparison to asymptomatic (median viremia: 1.7 × 10^4^ copies/mL vs. 2.0 × 10^3^ copies/mL (*p* = 0.002), median viruria: 1.0 × 10^7^ copies/mL versus 6.9 × 10^5^ copies/mL (*p* = 0.001), respectively).

**Conclusions:**

IL12B rs3212227 was associated with VL in cCMV. Symptomatic newborns had significantly higher viremia and viruria. The role of SNPs in pathogenesis of cCMV warrants further investigations.

## Introduction

Human cytomegalovirus (HCMV) causes the most common mother-to-a-child intrauterine infection. Congenital cytomegalovirus (cCMV) infection concerns 1% of newborns, which results in about 40,000 infected newborns in the United States each year.^1^ cCMV is diagnosed when HCMV is detected in newborn urine until 21st day of life by qualitative real-time polymerase chain reaction (PCR).^2^ Viral load (VL) in whole blood (viremia) and urine (viruria) is usually measured at diagnosis and during antiviral treatment in cCMV infection. Infants with cCMV infection have significantly higher viruria than infants with postnatal CMV (pCMV) infection, acquired through the birth canal or breast milk.^2^, ^3^ Moreover, women who transmit HCMV to neonate during prenatal period have higher HCMV DNA levels in urine, blood and amniotic fluid.^4^, ^5^ It has been suggested that higher VL could be a marker of the increased risk of significant neurological sequelae in cCMV infection.^6^ However, the real predictive value of VL for short- and long-term outcomes in cCMV infection is still unclear. There are limited data on factors that affect the level of viremia and viruria in cCMV-infected newborn infants. Monocytes, cytokines and pattern recognition receptors (PRRs) seem to be the first line of host immune defense against HCMV.^7^ Single nucleotide polymorphisms (SNPs) in genes encoding cytokines and cytokine receptors might participate in the complex interplay between the host immune system and HCMV and determine VL.^8^, ^9^, ^10^, ^11^, ^12^, ^13^, ^14^

This study aimed to evaluate association between SNPs in genes encoding cytokines and cytokine receptors (IL1B rs16944, IL12B rs3212227, IL28B rs12979860, CCL2 rs1024611, DC-SIGN rs735240, TLR2 rs5743708,TLR4 rs4986791, TLR9 rs352140) and viremia and viruria in newborn infants with cCMV infection.

## Materials and Methods

### Study population

Among 233 Polish (Caucasian) neonates (49.36% males) screened for cCMV infection, there were 92 newborn infants with confirmed cCMV infection (73 - symptomatic and 19 - asymptomatic) prospectively enrolled into the study in the Neonatal Intensive Care Unit (NICU) of The Children’s Memorial Health Institute in Warsaw between 2016 and 2019.^15^ Healthy control group consisted of 141 neonates with excluded cCMV infection.

Inclusion criteria were cCMV infection confirmed by qualitative HCMV DNA detection in urine sample collected until 21st day of life and parental consent. Exclusion criteria were multiple congenital anomalies, severe course of bacterial septic disease, other TORCH infections (e.g. congenital toxoplasmosis) and lack of parental consent.^15^ Clinical examination, neuroimaging [cranial ultrasonography (cUS); brain magnetic resonance imaging, (MRI)], audiological diagnostics [Otoacoustic Emission, (OAE); Auditory Brainstem Response (ABR)], ophthalmology consultation, laboratory blood tests, qualitative and quantitative real-time PCR DNA HCMV in blood and urine were performed during hospitalisation in the NICU. Symptomatic cCMV infection was diagnosed in newborn with at least one of the central nervous system abnormalities including microcephaly, sensorineural hearing loss (SNHL was defined as air conduction thresholds <20 dBHL on the ABR with normal bone conduction thresholds and normal middle ear function), chorioretinitis, abnormal neuroimaging finding in MRI (ventriculomegaly, white matter abnormalities, cerebral cortex defect, peri/intraventricular cyst, intracerebral calcifications, abnormal myelinisation), or at least three hepatobiliary and/or reticuloendothelial system disorders: hepatomegaly, splenomegaly, petechiae, cholestasis (direct bilirubin level <1 mg/dL), elevated liver aminotransferases (aspartate transaminase, ASPAT<84 U/L and/or alanine aminotransferase, ALTAT<60 U/L), neutropenia (neutrophils count <1000 K/μL), thrombocytopenia (platelets count <100 K/μL), anemia. Newborns with isolated intrauterine growth restriction (IUGR) or born prematurely (<37 weeks gestation) without other symptoms were not considered as symptomatic cases.^15^

### DNA isolation

Genomic DNA was extracted from 200 μL of whole blood and urine using a Nucleospin Tissue (Macherey-Nagel, Duren, Germany) on QIAcube instrument (Qiagen, Hilden, Germany) with final elution volume of 100 μL, according to the manufacturer’s instructions.

### Qualitative and quantitative detection of HCMV DNA

The presence of HCMV DNA was evaluated primarily in urine and blood by qualitative real-time PCR as described previously.^11^ Viremia and viruria were determined in HCMV DNA positive samples by using the Gene Proof Cytomegalovirus PCR Kit (Gene Proof, Brno, Czech Republic), which amplifies a region of gene encoding the 4 IE antigen. Amplification was performed according to the manufacturer’s instructions using a LightCycler 480 II Instrument (Roche, Indianapolis, IN). The results were expressed as copies/mL.

### Candidate genes selection

SNPs in eight candidate genes were selected *a priori* based on previously reported associations in HCMV infection.^8^, ^9^, ^10^, ^11^, ^12^, ^13^, ^14^ TLR-2 (Toll-like receptor), TLR-4, TLR-9 and DC-SIGN (dendritic cell-specific ICAM-grabbing non-integrin) were chosen for their role in HCMV recognition and triggering inflammatory cytokines.^10^, ^16^, ^17^, ^18^, ^19^, ^20^ IL-1B, IL-12B and IL-28B were chosen for their pro-inflammatory and immunomodulatory roles in the context of HCMV infection.^11^, ^13^, ^21^, ^22^ Finally, CCL2 (C-C motif chemokine ligand 2), a monocyte chemotactic protein-1 (MCP-1), was chosen due to its role in immune cell differentiation and their migration during inflammation in HCMV infection.^23^

### Determination of SNP genotypes

SNPs genotyping was performed using genomic DNA extracted from whole blood collected for qualitative real-time PCR reaction. TaqMan SNP Predesigned Genotyping Assays (Applied Biosystems, Inc., Foster City, CA, USA) were applied to a panel of eight polymorphisms (IL1B rs16944, IL12B rs3212227, IL28B rs12979860, CCL2 rs1024611, DC-SIGN rs735240, TLR2 rs5743708, TLR4 rs4986791 and TLR9 rs352140). The allelic discrimination was performed on the 7500 Real-time PCR System (Applied Biosystems) according to the manufacturer’s instructions. A blinded duplicated genotyping of 10 random study samples demonstrated 100% concordance.

### Statistical analysis

The normality of the distribution of analyzed data was assessed by Shapiro–Wilk test. Thus, data were presented as median and interquartile ranges (IQR). Non-parametric tests (Mann– Whitney and Kruskal–Wallis ANOVA) were used due to the non-normal distribution of analyzed variables. The categorical variables were presented as numbers and percentages, and Pearson’s Chi-squared test was used for comparisons. The association analysis was performed using SNP Stats software^24^ and STATISTICA10. The analysis of the association between eight selected SNPs and VL was calculated separately for cCMV-infected, symptomatic and asymptomatic newborn infants. All SNPs (cCMV-infected and healthy control groups) were analyzed for Hardy–Weinberg equilibrium (HWE) by using the Chi-square test. *p* ≤ 0.05 was considered significant.

## Results

### Study population characteristics

General maternal and neonatal characteristics of the study groups are presented in Table 1. Among cCMV-infected infants, there were 72 (78.26%) infants with abnormal cUS, 29 (35.80%) with abnormal hearing in any ear in ABR, 22 (23.91%) with microcephaly, 17 (18.48%) with chorioretinitis in at least one eye and finally 22 (23.91%) with at least three hepatobiliary and/or reticuloendothelial system disorders. Among all cCMV-infected newborn infants, median viremia was 1.0 × 10^4^ (IQR: 2.0 × 10^3^–9.8 × 10^4^) copies/mL, while median viruria was 8.7 × 10^6^ (IQR: 1.1 × 10^6^–1.0 × 10^7^) copies/ mL. Higher viremia was observed in symptomatic infants than in asymptomatic infants [median 1.7 × 10^4^ (IQR: 3.7 × 10^3^– 1.4 × 10^5^) copies/mL versus median 2.0 × 10^3^ (IQR: 9.9 × 10^2^– 4.0 × 10^3^) copies/mL; *p* = 0.002), respectively]. Similarly, higher viruria was observed in symptomatic than in asymptomatic neonates [median 1.0 × 10^7^ (IQR: 1.7 × 10^6^–1.0 × 10^7^) copies/ mL versus median 6.9 × 10^5^ (IQR: 1.4 × 10^5^–6.1 × 10^6^) copies/ mL; *p* = 0.001, respectively].

**Table 1:** Maternal and neonatal demographic data and general characteristics of cCMV-infected infants, with subgroups of symptomatic and asymptomatic infants.

Characteristics	cCMV-infected infants (*n* = 92)	Symptomatic infants (*n* = 73)	Asymptomatic infants (*n* = 19)	*p*-value^a^
Maternal age at delivery, (years)	28 (25–31)	29 (25–31)	28 (25–31)	NS
Primipara, *n* (%)	42 (45.65)	33 (45.21)	9 (47.37)	NS
Preterm delivery (<37Hbd), *n* (%)	23 (25.00)	19 (26.03)	4 (21.05)	NS
Caesarean section, *n* (%)	49 (54.44)	45 (61.64)	4 (21.05)	0.006
Prenatal HCMV screening, *n* (%)	37 (40.22)	27 (36.99)	10 (52.63)	NS
Birth weight, (g)	(21702755 –3200)	(21502685 –3140)	(26803050 –3630)	0.028
Gestational age, (weeks)	38 (37–39)	38 (37–39)	39 (37–40)	NS
Male, *n* (%)	46 (50.00)	36 (49.32)	10 (52.63)	NS
Age at admission to NICU, Median (IQR), (days)	16 (9–23)	14 (9–21)	22 (20–35)	<0.001
Weight at admission to NICU, (g)	3040 (2440–3450)	2980 (2340–3320)	3600 (2930–4310)	<0.001
Age at first HCMV DNA detection in urine, (days)	9 (4–15)	8 (3–14)	17 (8–20)	0.002

Data are presented as median (IQR) or number (%). cCMV, congenital cytomegalovirus; HCMV, human cytomegalovirus; IQR, interquartile range; NICU, Neonatal Intensive Care Unit.

a *p*-value for comparison between symptomatic and asymptomatic infants.

### Distribution of SNP genotypes

Genotype distribution of examined SNPs was obtained in HWE in the entire study population (comparison between healthy control group and cCMV-infected infants as well as symptomatic and asymptomatic infants). We found no significant differences in genotype distribution of assessed SNPs between symptomatic and asymptomatic newborn infants (Table S1 in Supplementary Materials). Results of genotype distribution have been previously presented for cCMV-infected and healthy control group and also at least partially for symptomatic and asymptomatic cases^15^

### Frequencies of SNP alleles

Allele frequencies of examined SNPs were obtained in HWE in the entire study population (comparison between healthy control group and cCMV-infected infants as well as symptomatic and asymptomatic infants) (Tables S2–S3 in Supplementary Materials). No significant differences were found in allele frequencies of assessed SNPs in both above configurations.

### Association between SNP genotypes and VL in blood and urine

**Figure 1:**
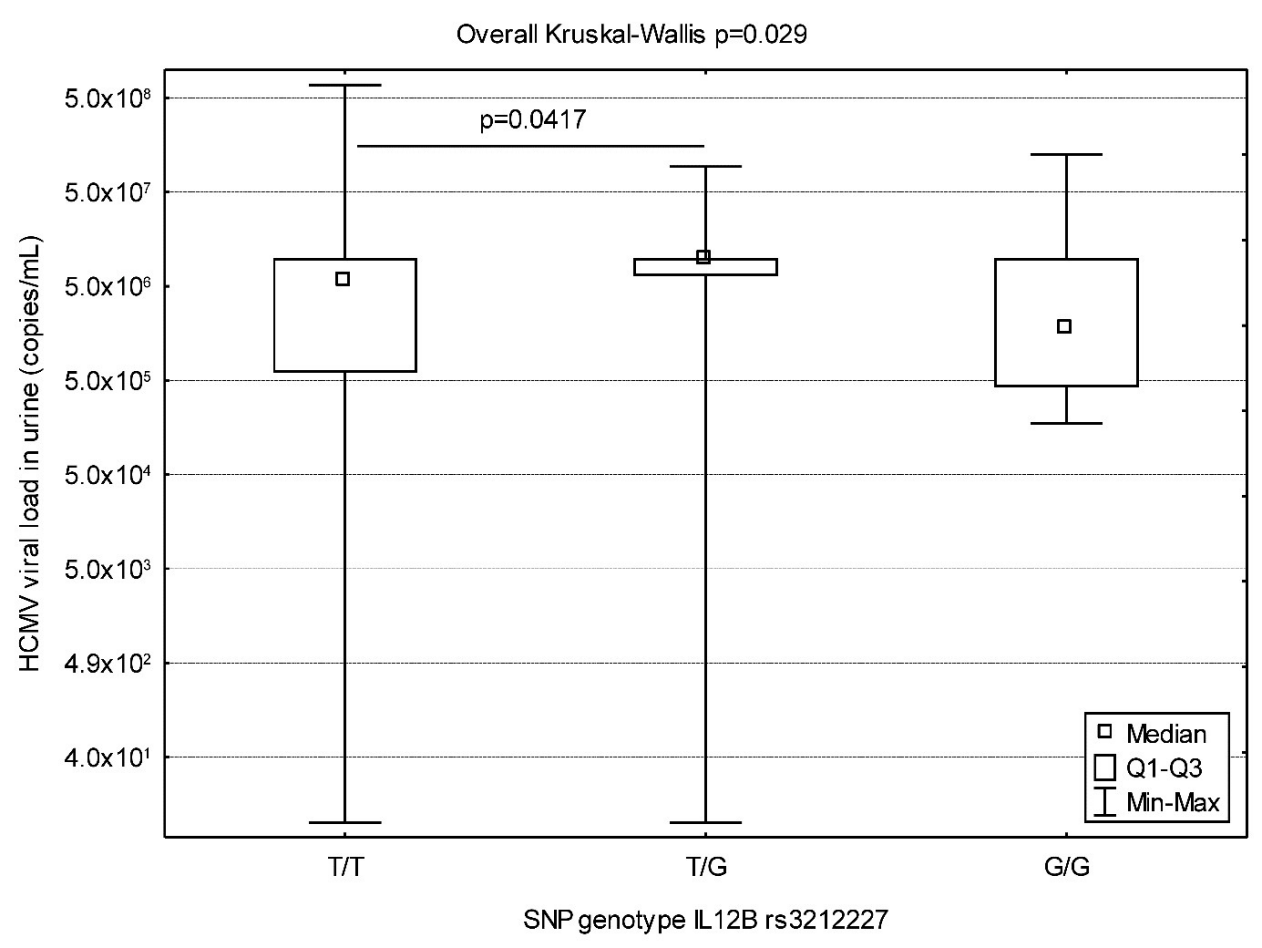
HCMV VL in urine depending on genotypes of IL12B rs3212227 polymorphism. Bars in the box show the first and the third quartiles. Whisker charts represent the minimum to maximum values. Small box represents median value. *p*-value below 0.05 is statistically significant. Kruskal-Wallis ANOVA test was used. HCMV: human cytomegalovirus; VL: viral load.

First, we analyzed the association between eight examined SNPs and VL in blood and urine in 92 neonates with cCMV infection. In the overall analysis, only the association between IL12B rs3212227 polymorphism and viruria was observed (*p* = 0.029; Table 2). Then, in multiple comparison tests in relation to IL12B rs3212227 polymorphism, we found the association between two of three genotypes: infants carrying heterozygous T/G genotype were promoted to have higher viruria than T/T genotype (*p* = 0.041; Figure 1). Although neonates with G/G genotype had the lowest number of viral copies in urine, this result did not reach statistical significance in relation to the other two genotypes in this polymorphism (*p* = 0.305 for association between G/G and T/G and *p* = 1.000 for association between G/G and T/T). We also found no associations between examined SNPs and viremia and viruria in both study subgroups, either symptomatic or asymptomatic infants (Tables S4–S5 in Supplementary Materials).

**Table 2:** Association between genotypes of examined polymorphisms (SNPs) and viremia and viruria in newborn infants with cCMV infection.

SNP, rs number^a^, alleles	Genotype	n = 92, n (*%*)	HCMV DNA (copies/mL) in blood	*p-value* ^b^	HCMV DNA (copies/mL) in urine	*p-value* ^c^
IL1B	A/A	7 (7.6)	6.3 × 10^3^ (3.8 × 10^3^–2.7 × 10^4^)		6.5 × 10^6^ (2.8 × 10^6^–1.0 × 10^7^)	
rs16944	A/G	49 (53.3)	8.6 × 10^3^ (1.9 × 10^3^–5.8 × 10^4^) (*n* = 48)	NS	7.8 × 10^6^ (5.1 × 10^5^–1.0 × 10^7^) (*n* = 48)	NS
G/A	G/G	36 (39.1)	1.5 × 10^4^ (2.0 × 10^3^–2.0 × 10^5^) (*n* = 34)		1.0 × 10^7^ (1.7 × 10^6^–1.0 × 10^7^) (*n* = 35)	
IL12B	T/T	57 (62.0)	2.2 × 10^4^ (2.1 × 10^3^–1.5 × 10^5^) (*n* = 54)		5.8 × 10^6^ (6.0 × 10^5^–1.0 × 10^7^) (*n* = 56)	
rs3212227	T/G	28 (30.4)	7.4 × 10^3^ (1.8 × 10^3^–4.7 × 10^4^)	NS	1.0 × 10^7^ (6.4 × 10^6^–1.0 × 10^7^) (*n* = 27)	0.029
G/T	G/G	7 (7.6)	4.3 × 10^3^ (2.1 × 10^3^–3.0 × 10^4^)		1.7 × 10^6^ (4.2 × 10^5^–1.0 × 10^7^)	
IL28B	T/T	13 (14.1)	5.8 × 10^3^ (1.9 × 10^3^–1.3 × 10^5^) (*n* = 12)		2.8 × 10^6^ (4.0 × 10^5^–1.0 × 10^7^) (*n* = 12)	
rs12979860	T/C	38 (41.3)	1.0 × 10^4^ (1.6 × 10^3^–1.3 × 10^5^)	NS	1.0 × 10^7^ (2.8 × 10^6^–1.0 × 10^7^) (*n* = 37)	NS
C/T	C/C	41 (44.6)	1.2 × 10^4^ (2.1 × 10^3^–8.2 × 10^4^) (*n* = 39)		8.6 × 10^6^ (8.4 × 10^5^–1.0 × 10^7^)	
CCL2	G/G	3 (3.3)	2.4 × 10^3^ (0–9.4 × 10^3^)		1.0 × 10^7^ (9.2 × 10^6^–1.0 × 10^7^)	
rs1024611	G/A	39 (42.4)	1.7 × 10^4^ (1.7 × 10^3^–5.8 × 10^4^) (*n* = 38)	NS	5.5 × 10^6^ (5.2 × 10^5^–1.0 × 10^7^) (*n* = 37)	NS
A/G	A/A	50 (54.3)	8.9 × 10^3^ (2.4 × 10^3^–1.4 × 10^5^) (*n* = 48)		8.5 × 10^6^ (1.6 × 10^6^–1.0 × 10^7^)	
DC-SIGN	G/G	35 (38.0)	1.2 × 10^4^ (1.4 × 10^3^–1.2 × 10^5^) (*n* = 32)		1.0 × 10^7^ (1.3 × 10^6^–1.0 × 10^7^)	
rs735240	G/A	37 (40.2)	1.1 × 10^4^ (2.4 × 10^3^–1.7 × 10^5^)	NS	7.6 × 10^6^ (1.1 × 10^6^–1.0 × 10^7^) (*n* = 36)	NS
A/G	A/A	20 (21.7)	7.4 × 10^3^ (1.7 × 10^3^–3.5 × 10^4^)		5.5 × 10^6^ (5.0 × 10^5^–1.0 × 10^7^) (*n* = 19)	
TLR2	G/G	82 (89.1)	1.2 × 10^4^ (2.1 × 10^3^–1.1 × 10^5^) (*n* = 79)		7.8 × 10^6^ (1.2 × 10^6^–1.0 × 10^7^) (*n* = 80)	
rs5743708	G/A	10 (10.9)	4.3 × 10^3^ (1.6 × 10^3^–3.8 × 10^4^)	NS	1.0 × 10^7^ (1.0 × 10^6^–1.0 × 10^7^)	NS
A/G	A/A	0 (0)	NA		NA	
TLR4	T/T	0 (0)	NA		NA	
rs4986791	T/C	9 (9.8)	2.0 × 10^4^ (2.8 × 10^3^–9.8 × 10^4^)	NS	1.0 × 10^7^ (1.7 × 10^6^–1.0 × 10^7^)	NS
C/T	C/C	83 (90.2)	1.0 × 10^4^ (2.0 × 10^3^–1.0 × 10^5^) (*n* = 80)		8.6 × 10^6^ (1.1 × 10^6^–1.0 × 10^7^) (*n* = 81)	
TLR9	T/T	30 (32.6)	2.4 × 10^4^ (3.6 × 10^3^–2.6 × 10^5^) (*n* = 29)		1.0 × 10^7^ (1.7 × 10^6^–1.0 × 10^7^) (*n* = 29)	
rs352140	T/C	47 (51.1)	8.4 × 10^3^ (2.1 × 10^3^–5.7 × 10^4^) (*n* = 45)	NS	5.7 × 10^6^ (8.4 × 10^5^–1.0 × 10^7^) (*n* = 46)	NS
C/T	C/C	15 (16.3)	7.9 × 10^3^ (1.1 × 10^3^–3.7 × 10^4^)		1.0 × 10^7^ (1.6 × 10^6^–1.0 × 10^7^)	

Data are presented as median (IQR) or number (%). NS, not significant (*p* < 0.05). cCMV, congenital cytomegalovirus; CCL2,C-C motif chemokine ligand 2; DC-SIGN, dendritic cell-specific ICAM-grabbing non-integrin; HCMV, human cytomegalovirus; IL, interleukin; IQR, interquartile range; NA, not applicable; SNP, single nucleotide polymorphism; TLR, toll-like receptor.

a SNP database (dbSNP) reference number (ID number).

b *p*-value – for comparison between genotypes and viremia.

c *p*-value – for comparison between genotypes and viruria.

### Association between SNP alleles and VL in blood and urine

We analyzed the association between alleles of eight examined SNPs and VL in blood and urine in 92 neonates with cCMV infection including 73 symptomatic and 19 asymptomatic infants. We observed the association between T allele of IL12B rs3212227 polymorphism and higher viremia in symptomatic infants only (*p* = 0.037; Table 3). No other associations between alleles and VL were found (Tables S6– S7 in Supplementary Materials)

**Table 3:** Association between alleles of examined polymorphisms (SNPs) and viremia and viruria in symptomatic newborn infants with cCMV infection.

Symptomatic newborn infants with cCMV infection (*N* = 73)						
SNP, rs number^a^, alleles	Allele	Alleles, *n* (%)	HCMV (copies/DNA mL) in blood	*p*-value^b^	HCMV DNA (copies/mL) in urine	*p*-value^c^
IL1B	G	97 (66.44)	2.2 × 10^4^ (3.0 × 10^3^–1.9 × 10^5^) (*n* = 96)		1.0 × 10^7^ (2.9 × 10^6^–1.0 × 10^7^) (*n* = 94)	
rs16944				NS		NS
G/A	A	49 (33.56)	1.1 × 10^4^ (3.8 × 10^3^–5.4 × 10^4^) (*n* = 48)		1.0 × 10^7^ (1.5 × 10^6^–1.0 × 10^7^) (*n* = 48)	
IL12B	T	110 (75.34)	2.6 × 10^4^ (4.7 × 10^3^–1.8 × 10^5^)		1.0 × 10^7^ (1.4 × 10^6^–1.0 × 10^7^)	
rs3212227			(*n* = 108)	0.037	(*n* = 107)	NS
G/T	G	36 (24.66)	9.4 × 10^3^ (2.4 × 10^3^–3.4 × 10^4^)		1.0 × 10^7^ (1.7 × 10^6^–1.0 × 10^7^) (*n* = 35)	
IL28B	C	92 (63.01)	1.7 × 10^4^ (3.8 × 10^3^–1.2 × 10^5^)		1.0 × 10^7^ (1.7 × 10^6^–1.0 × 10^7^) (*n* = 91)	
rs12979860				NS		NS
C/T	T	54 (36.99)	1.9 × 10^4^ (3.3 × 10^3^–1.8 × 10^5^) (*n* = 52)		1.0 × 10^7^ (1.6 × 10^6^–1.0 × 10^7^) (*n* = 51)	
						
CCL2	A	110 (75.34)	1.9 × 10^4^ (3.9 × 10^3^–1.7 × 10^5^)		1.0 × 10^7^ (1.7 × 10^6^–1.0 × 10^7^)	
rs1024611			(*n* = 108)	NS	(*n* = 108)	NS
A/G	G	36 (24.66)	1.6 × 10^4^ (2.0 × 10^3^–5.4 × 10^4^)		1.0 × 10^7^ (1.4 × 10^6^–1.0 × 10^7^) (*n* = 34)	
DC-SIGN	G	87 (59.59)	2.8 × 10^4^ (3.9 × 10^3^–1.5 × 10^5^) (*n* = 85)		1.0 × 10^7^ (2.9 × 10^6^–1.0 × 10^7^) (*n* = 86)	
rs735240				NS		NS
A/G	A	59 (40.41)	1.2 × 10^4^ (3.3 × 10^3^–8.2 × 10^4^)		1.0 × 10^7^ (1.5 × 10^6^–1.0 × 10^7^) (*n* = 56)	
TLR2	G	138 (94.52)	1.7 × 10^4^ (3.7 × 10^3^–1.5 × 10^5^)		1.0 × 10^7^ (1.7 × 10^6^–1.0 × 10^7^)	
rs5743708			(*n* = 136)	NS	(*n* = 134)	NS
A/G	A	8 (5.48)	5.0 × 10^3^ (2.8 × 10^3^–8.4 × 10^4^)		1.0 × 10^7^ (5.5 × 10^6^–1.0 × 10^7^)	
TLR4 rs4986791	C	139 (95.21)	1.7 × 10^4^ (3.8 × 10^3^–1.5 × 10^5^) (*n* = 137)	NS	1.0 × 10^7^ (1.7 × 10^6^–1.0 × 10^7^) (*n* = 135)	NS
C/T	T	7 (4.79)	3.8 × 10^4^ (2.8 × 10^3^–1.1 × 10^5^)		1.0 × 10^7^ (1.7 × 10^6^–1.0 × 10^7^)	
TLR9	T	84 (57.53)	2.8 × 10^4^ (3.8 × 10^3^–1.9 × 10^5^) (*n* = 83)		1.0 × 10^7^ (2.9 × 10^6^–1.0 × 10^7^) (*n* = 81)	
rs352140				NS		NS
C/T	C	62 (42.47)	9.4 × 10^3^ (2.8 × 10^3^–5.8 × 10^4^) (*n* = 61)		1.0 × 10^7^ (1.6 × 10^6^–1.0 × 10^7^) (*n* = 61)	

Data are presented as median (IQR) or number (%). cCMV, congenital cytomegalovirus; CCL2, C-C motif chemokine ligand 2; DC-SIGN, dendritic cell-specific ICAM-grabbing non-integrin; HCMV, human cytomega lovirus; IL, interleukin; IQR, interquartile range; NS, not significant (*p* < 0.05); SNP, single nucleotide polymorphism; TLR, toll-like receptor.

a SNP database (dbSNP) reference number (ID number).

b *p*-value – for comparison between alleles and viremia.

c *p*-value – for comparison between alleles and viruria.

## Discussion

This prospective study investigated associations between eight SNPs in genes encoding cytokines and cytokine receptors (IL1B rs16944, IL12B rs3212227, IL28B rs12979860, CCL2 rs1024611, DC-SIGN rs735240, TLR2 rs5743708, TLR4 rs4986791 and TLR9 rs352140) and VL in blood and urine in newborn infants with cCMV infection.

We found the novel associations between IL12B polymorphism and VL in neonates with cCMV infection. We observed that heterozygous T/G genotype of IL12B rs3212227 polymorphism was associated with higher viruria than T/T genotype in cCMV-infected neonates. The strength of these results was not supported by alleles analysis. Neither T nor G allele of IL12B rs3212227 promoted higher viruria separately. We assume that probably only heterozygous T/G status (both alleles altogether) uphold increased HCMV DNA concentration in urine. Interestingly, T allele of IL12B rs3212227 was associated with higher viremia in symptomaticneonates. However, neither T/T nor T/G genotype was connected with higher VL in blood as could be expected.

Popescu et al. proved that impaired CMV-specific T helper (Th) cellular immunity is highly dependent on IL-12.^25^ IL-12 is an inflammatory cytokine that initiates T-cell diferentiation towards Th1 and inhibits Th2 lymphocyte activity.^26^ So far, SNPs of IL-12 were found to be associated with other viral infections, such as hepatitis B virus (HBV), hepatitis C virus (HCV) and also with HCMV infection among adult patients.^27^, ^28^, ^29^ Seegers et al. link IL12 rs3212227 SNP polymorphism with increased IL-12 production.^30^ We only might speculate that newborn infants carrying heterozygous T/G genotype of IL12B rs3212227 produce higher amount of IL-12. Presumably, IL-12 could promote higher urinary HCMV excretion to eliminate the virus. Our study might support a hypothesis on the protective role of IL12 polymorphism in a host defense against HCMV infection. However, we could not explain contradictory data of T allele and T-linked genotypes in relation to viremia. It should be underlined that the number of newborns in each subgroup was relatively small, despite the fact, it was one of the largest population of cCMV-infected neonates that was genetically studied so far. Certainly, further investigations are warranted to confirm the association between SNP of IL12B rs3212227 and higher viruria and verify the role of T allele in HCMV DNA concentration in blood.

There is still no consensus whether viruria is a result of kidney virus filtering during viremia or HCMV origins from infected renal cells. HCMV kidney tropism was widely documented, mostly among renal allograft recipients.^31^ In relation to cCMV infection, the highest amount of HCMV was observed in pancreas, liver, kidney and brain of the fetus, which suggested a disseminated HCMV infection.^32^ On the other hand, cCMV-infected infants tend to excrete virus in the urine for even 3–5 years,^33^ even if HCMV is not detected in the blood. Our results may support previous findings showing that viruria might be less likely to be secondary to HCMV DNA concentration in blood than to renal cells infection.

Symptomatic infants with cCMV infection excrete higher amount of HCMV DNA in urine than asymptomatic infants.^6^ We also observed significantly higher viruria and viremia in symptomatic infants. It should be underlined that higher VL can be linked to an increased risk of unfavourable sequelae of cCMV infection.^34^, ^35^ Smiljkovic et al. showed that infant would have <75% probability of being symptomatic above 18,770 copies/mL, with a threshold of 100,000 copies/ mL approaching a 100% probability.^36^ On the other hand, according to Marsico et al., majority of newborns on antiviral therapy without viral suppression still had improved hearing.^37^ The predictive role of the VL and unfavourable sequelae in infants with cCMV infection is still questionable and needs further investigations.^6^, ^38^

There are limited data on the associations between SNPs and VL in cCMV infection. Most of them concerned SNPs of TLRs. Paradowska et al. documented that heterozygous variant of TLR9-1486T<C (rs187084) was associated with statistically higher HCMV DNA concentration in blood in HCMV-infected infants.^18^ Studzińska et al. reported higher viremia and viruria and increased risk of symptomatic HCMV infection in infants with heterozygous genotype of TLR3 rs3775291. Higher HCMV viremia was also noted in heterozygous genotype of TLR7 rs5741880.^39^ However, above-mentioned reports included both children with cCMV and pCMV infection. As far as cCMV infection was concerned, no associations were found between TLR2 2258 G<A SNP (rs5743708) and HCMV VL in neonates, but G/A variants in TLR2 2258 and TLR9 2848 C<T (rs352140) SNPs were correlated with higher VLs in fetal amniotic fluid and maternal urine samples.^19^ In our study, none of three examined TLR SNPs (TLR2 rs5743708, TLR4 rs4986791, TLR9 rs352140) were associated with higher viremia or viruria. We also found no association between CCL2 and DC-SIGN SNPs and VL in blood and urine.

We compared allele frequencies of our study population to the reference population from the Reference SNP Report.^40^ As we expected, our study population allele frequencies were very similar to the frequencies in the reference European population (the difference level did not exceed 5 percentage points) (Table S1 in Supplementary Materials).

Some limitations of our study should be acknowledged. We are aware that there are some confounding factors which may affect the results, but the current study was not designed to address these issues. First, the genetic background and the serological status of the mother are relevant for the risk of primary infection. Undoubtedly, the type of maternal infection (primary or non-primary) and the time of fetal infection may impact the genetic study results. Moreover, the diversity of HCMV strains may afect the results of the study. Although the current study population is larger than in our previous report,^11^ the sample size might be still too small for genetic associations. In addition, no correction for multiple testing was applied, and so type I error cannot be completely ruled out.

The important strength of our study is its prospective design. Furthermore, we included homogeneous population consisted of Caucasian central European newborn infants with cCMV infection, what let us eliminate potential race dependent genetic factors. We focused on congenital infection only because we thought that diferent genetic mechanisms might determine congenital and postnatal HCMV infection. What is more, five of eight selected SNPs were analyzed for the first time in the context of cCMV infection.^15^ To the best of our knowledge, this is the first and the largest study that analyzed the association between the eight SNPs and VL, and the first study that suggests the novel association between IL-12B polymorphism and VL in cCMV infection. However, future studies are necessary to replicate these findings in the larger study population.

To summarise, we found the novel associations between IL-12B polymorphism and VL in cCMV-infected newborns. Our results confirmed that symptomatic newborn infants had higher viremia and viruria than asymptomatic newborn infants. Indisputably, the role of SNPs in the pathogenesis of cCMV requires further investigations, which may change prognosis for newborn infants with cCMV in the future.

## Key points

SNPs in genes encoding cytokines and cytokine receptors might determine the level of VL in cCMV infection.

Association between eight SNPs of IL1B, IL12B, IL28B, CCL2, DC-SIGN, TLR2 and TLR4, TLR9 and VL in cCMV infection was studied.

T/G genotype of IL12B rs3212227 was associated with higher viruria than T/T genotype in cCMV infection.

T allele of IL12B rs3212227 was associated with higher viremia in comparison to G allele in symptomatic cCMV infection. Symptomatic newborns had significantly higher viremia and viruria.

No other associations between SNPs and VL were reported in newborns with cCMV-infection.

SNPs analysis might be suitable for better identification and diagnosis of newborns (or even fetuses) with cCMV infection.

## Figures and Tables

**Table S1:** Distribution of genotypes of examined SNPs in the study population of cCMV-infected infants and subgroups of symptomatic and asymptomatic newborn infants

SNP, rs number^a^, alleles	Genotype	cCMV-infected neonates(*n* = 92)	Symptomatic neonates (*n* = 73)	Asymptomatic neonates (*n* = 19)	*p*-value^b^

			Genotype frequencies, *n* (%)		
IL1B	A/A	7 (7.61)	5 (6.85)	2 (10.53)	
rs16944	A/G	49 (53.26)	39 (53.42)	10 (52.63)	NS
G/A	G/G	36 (39.13)	29 (39.73)	7 (36.84)	
IL12B	T/T	57 (61.96)	43 (58.90)	14 (73.68)	
rs3212227	T/G	28 (30.43)	24 (32.88)	4 (21.05)	NS
G/T	G/G	7 (7.61)	6 (8.22)	1 (5.26)	
IL28B	T/T	13 (14.13)	11 (15.07)	2 (10.53)	
rs12979860	T/C	38 (41.30)	32 (43.84)	6 (31.58)	NS
C/T	C/C	41 (44.57)	30 (41.09)	11 (57.89)	
CCL2	G/G	3 (3.26)	3 (4.11)	0 (0.00)	
rs1024611	G/A	39 (42.39)	30 (41.10)	9 (47.37)	NS
A/G	A/A	50 (54.35)	40 (54.79)	10 (52.63)	
DC-SIGN	G/G	35 (38.04)	28 (38.36)	7 (36.84)	
rs735240	G/A	37 (40.22)	31 (42.47)	6 (31.58)	NS
A/G	A/A	20 (21.74)	14 (19.17)	6 (31.58)	
TLR2	G/G	82 (89.13)	65 (89.04)	17 (89.47)	
rs5743708	G/A	10 (10.87)	8 (10.96)	2 (10.53)	NS
A/G	A/A	0 (0.00)	0 (0.00)	0 (0.00)	
TLR4	T/T	0 (0.00)	0 (0.00)	0 (0.00)	
rs4986791	T/C	9 (9.78)	7 (9.59)	2 (10.53)	NS
C/T	C/C	83 (90.22)	66 (90.41)	17 (89.47)	
TLR9	T/T	30 (32.61)	23 (31.51)	7 (36.84)	
rs352140	T/C	47 (51.09)	38 (52.05)	9 (47.37)	NS
C/T	C/C	15 (16.30)	12 (16.44)	3 (15.79)	

Data are presented as number (%). cCMV, congenital cytomegalovirus; *CCL2*,C-C *motif*chemokine ligand 2; DC-SIGN, dendritic cell-specific ICAM-grabbing non-integrin; IL*, interleukin;* NS, not significant (*p* < 0.05); SNP, single nucleotide polymorphism; TLR, toll-like receptor.

a SNP database (dbSNP) reference number (ID number).

b *p*-value for comparison between frequencies of genotypes and subgroups of symptomatic and asymptomatic newborn infants.

**Table S2:** Allele frequencies of examined SNPs in the study population in comparison to known European and Global reference populations from the Reference SNP (rs) Report

SNP,rs number^a^, alleles	Allele	All study population (*n* = 233)	Uninfected control infants (*n* = 141)	cCMV-infected infants (*n* = 92)	*p*-value^b^	Reference population^c^	
						European	Global
		Allele frequencies, *n* (%)				Allele frequencies, %	
IL1B	G	320 (68.67)	199 (70.57)	121 (65.76)		65.01	49.06
rs16944					NS		
G/A	A	146 (31.33)	83 (29.43)	63 (34.24)		34.99	50.94
IL12B	T	352(75.54)	210 (74.47)	142 (77.17)		77.73	64.10
rs3212227					NS		
G/T	G	114 (24.46)	72 (25.53)	42 (22.83)		22.27	35.90
IL28B	C	309 (66.31)	189 (67.02)	120 (65.22)		69.09	64.42
rs12979860					NS		
C/T	T	157 (33.69)	93 (32.98)	64 (34.78)		30.91	35.58
CCL2	A	344 (73.82)	205 (72.70)	139 (75.54)		68.39	63.64
rs1024611					NS		
A/G	G	122 (26.18)	77(27.30)	45 (24.46)		31.61	36.36
DC-SIGN	G	278 (59.66)	171 (60.64)	107 (58.15)		56.76	64.70
rs735240					NS		
A/G	A	188 (40.34)	111 (39.36)	77 (41.85)		43.24	35.30
TLR2	G	441 (94.64)	267 (94.68)	174 (94.57)		97.61	99.32
rs5743708					NS		
A/G	A	25 (5.36)	15 (5.32)	10 (5.43)		2.39	0.68
TLR4	C	440 (94.42)	265 (93.97)	175 (95.11)		94.23	95.93
rs4986791					NS		
C/T	T	26 (5.58)	17 (6.03)	9 (4.89)		5.77	4.07
TLR9	T	269 (57.73)	162 (57.45)	107 (58.15)		54.57	41.55
rs352140					NS		
C/T	C	197 (42.27)	120 (42.55)	77 (41.85)		45.43	58.45

Data are presented as number (%). cCMV, congenital cytomegalovirus; *CCL2*, C-C *motif*chemokine ligand 2; DC-SIGN, dendritic cell-specific ICAM-grabbing non-integrin; IL*, interleukin;* NS, not significant (*p* < 0.05); SNP, single nucleotide polymorphism; TLR, toll-like receptor.

a SNP database (dbSNP) reference number (ID number).

b *p*-value for comparison between frequencies of alleles and subgroups of uninfected controls and CMV-infected newborn infants.

c Reference allele frequency based on 1000Genomes study from the Reference SNP (rs) Report.

**Table S3:** Allele frequencies of examined SNPs in the study population of cCMV-infected neonates and subgroups of symptomatic and asymptomatic neonates

SNP,rs number^a^, alleles	Allele	cCMV-infected infants (*n* = 92)	Symptomatic infants (*n* = 73)	Asymptomatic infants (*n* = 19)	*p*-value^b^

		Allele frequencies, *n* (%)			
IL1B	G	121 (65.76)	97 (66.44)	24 (63.16)	
rs16944					NS
G/A	A	63 (34.24)	49 (33.56)	14 (36.84)	
IL12B	T	142 (77.17)	110 (75.34)	32 (84.21)	
rs3212227					NS
G/T	G	42 (22.83)	36 (24.66)	6 (15.79)	
IL28B	C	120 (65.22)	92 (63.01)	28 (73.68)	
rs12979860					NS
C/T	T	64 (34.78)	54 (36.99)	10 (26.32)	
CCL2	A	139 (75.54)	110 (75.34)	29 (76.32)	
rs1024611					NS
A/G	G	45 (24.46)	36 (24.66)	9 (23.68)	
DC-SIGN	G	107 (58.15)	87 (59.59)	20 (52.63)	
rs735240					NS
A/G	A	77 (41.85)	59 (40.41)	18 (47.37)	
TLR2	G	174 (94.57)	138 (94.52)	36 (94.74)	
rs5743708					NS
A/G	A	10 (5.43)	8 (5.48)	2 (5.26)	
TLR4	C	175 (95.11)	139 (95.21)	36 (94.74)	
rs4986791					NS
C/T	T	9 (4.89)	7 (4.79)	2 (5.26)	
TLR9	T	107 (58.15)	84 (57.53)	23 (60.53)	
rs352140					NS
C/T	C	77 (41.85)	62 (42.47)	15 (39.47)	

Data are presented as number (%). cCMV, congenital cytomegalovirus; *CCL2*, C-C*motif*chemokine ligand 2; DC-SIGN, dendritic cell-specific ICAM-grabbing non-integrin; IL*, interleukin*; NS, not significant (*p* < 0.05); SNP, single nucleotide polymorphism;; TLR, toll-like receptor.

a SNP database (dbSNP) reference number (ID number).

b *p*-value for comparison between frequencies of alleles and subgroups of symptomatic and asymptomatic CMV-infected newborn infants.

**Table S4:** Association between genotypes of examined polymorphisms and viremia and viruria in symptomatic infants with cCMV infection

Symptomatic infants with cCMV infection (*n* = 73)						
SNP rs number^a^ alleles	Genotype	*n* = 73 *n* (%)	HCMV DNA (copies/mL) in blood	*p*-value^b^	HCMV DNA (copies/mL) in urine	*p*-value^c^
	A/A	5 (6.85)	6.3 × 10^3^ (5.4 × 10^3^–1.1 × 10^4^)		6.5 × 10^6^ (5.4 × 10^6^–1.0 × 10^7^)	
IL1B						
rs16944	A/G	39 (53.42)	1.9 × 10^4^ (3.6 × 10^3^–1.1 × 10^5^) (*n* = 38)	NS	1.0 × 10^7^ (1.1 × 10^6^–1.0 × 10^7^) (*n* = 38)	NS
G/A						
	G/G	29 (39.73)	3.0 × 10^4^ (2.8 × 10^3^–2.1 × 10^5^)		1.0 × 10^7^ (3.7 × 10^6^–1.0 × 10^7^) (*n* = 28)	
	T/T	43 (58.90)	3.9 × 10^4^ (5.4 × 10^3^–1.9 × 10^5^) (*n* = 42)		1.0 × 10^7^ (1.3 × 10^6^–1.0 × 10^7^) (*n* = 42)	
IL12B						
rs3212227	T/G	24 (32.88)	9.4 × 10^3^ (2.4 × 10^3^–4.7 × 10^4^)	NS	1.0 × 10^7^ (9.2 × 10^6^–1.0 × 10^7^) (*n* = 23)	NS
G/T						
	G/G	6 (8.22)	7.9 × 10^3^ (2.4 × 10^3^–3.0 × 10^4^)		2.3 × 10^6^ (1.6 × 10^6^–1.0 × 10^7^)	
	T/T	11 (15.07)	2.2 × 10^4^ (3.3 × 10^3^–2.1 × 10^5^) (*n* = 10)		7.0 × 10^6^ (1.3 × 10^6^–1.0 × 10^7^) (*n* = 10)	
IL28B						
rs12979860	T/C	32 (43.84)	1.9 × 10^4^ (3.2 × 10^3^–1.4 × 10^5^)	NS	1.0 × 10^7^ (3.5 × 10^6^–1.0 × 10^7^) (*n* = 31)	NS
C/T						
	C/C	30 (41.09)	1.6 × 10^4^ (3.9 × 10^3^–1.1 × 10^5^)		1.0 × 10^7^ (1.4 × 10^6^–1.0 × 10^7^)	
	G/G	3 (4.11)	2.4 × 10^3^ (0–9.4 × 10^3^)		1.0 × 10^7^ (9.2 × 10^6^–1.0 × 10^7^)	
CCL2						
rs1024611	G/A	30 (41.10)	2.9 × 10^4^ (3.6 × 10^3^–1.1 × 10^5^)	NS	1.0 × 10^7^ (1.0 × 10^6^–1.0 × 10^7^) (*n* = 28)	NS
A/G						
	A/A	40 (54.79)	1.2 × 10^4^ (3.9 × 10^3^–2.0 × 10^5^) (*n* = 39)		1.0 × 10^7^ (3.7 × 10^6^–1.0 × 10^7^)	
	G/G	28 (38.36)	2.9 × 10^4^ (4.3 × 10^3^–1.5 × 10^5^) (*n* = 27)		1.0 × 10^7^ (4.2 × 10^6^–1.0 × 10^7^)	
DC-SIGN						
rs735240	G/A	31 (42.47)	1.2 × 10^4^ (3.6 × 10^3^–1.9 × 10^5^)	NS	1.0 × 10^7^ (1.4 × 10^6^–1.0 × 10^7^) (*n* = 30)	NS
A/G						
	A/A	14(19.17)	1.3 × 10^4^ (2.8 × 10^3^–3.8 × 10^4^)		1.0 × 10^7^ (1.7 × 10^6^–1.0 × 10^7^) (*n* = 13)	
	G/G	65 (89.04)	1.9 × 10^4^ (3.7 × 10^3^–1.7 × 10^5^) (*n* = 64)		1.0 × 10^7^ (1.7 × 10^6^–1.0 × 10^7^) (*n* = 63)	
TLR2						
rs5743708	G/A	8 (10.96)	5.0 × 10^3^ (2.8 × 10^3^–8.4 × 10^4^)	NS	1.0 × 10^7^ (5.5 × 10^6^–1.0 × 10^7^)	NS
A/G						
	A/A	0 (0.00)	NA		NA	
	T/T	0 (0.00)	NA		NA	
TLR4						
rs4986791	T/C	7 (9.59)	3.8 × 10^4^ (2.8 × 10^3^–1.1 × 10^5^)	NS	1.0 × 10^7^ (1.7 × 10^6^–1.0 × 10^7^)	NS
C/T						
	C/C	66 (9×0.41)	1.4 × 10^4^ (3.8 × 10^3^–1.5 × 10^5^) (*n* = 65)		1.0 × 10^7^ (1.7 × 10^6^–1.0 × 10^7^) (*n* = 64)	
TLR9	T/T	23 (31.51)	2.9 × 10^4^ (5.3 × 10^3^–2.6 × 10^5^)		1.0 × 10^7^ (5.5 × 10^6^–1.0 × 10^7^) (*n* = 22)	
rs352140	T/C	38 (52.05)	1.4 × 10^4^ (2.8 × 10^3^–8.2 × 10^4^) (*n* = 37)	NS	1.0 × 10^7^ (1.1 × 10^6^–1.0 × 10^7^) (*n* = 37)	NS
C/T	C/C	12 (16.44)	8.6 × 10^3^ (3.0 × 10^3^–4.7 × 10^4^)		1.0 × 10^7^ (4.3 × 10^6^–1.0 × 10^7^)	

Data are presented as median (IQR- interquartile range) or number (%). cCMV, congenital cytomegalovirus; *CCL2*, C-C *motif* chemokine ligand 2; DC-SIGN, dendritic cell-specific ICAM-grabbing non-integrin; HCMV, human cytomegalovirus; IL*, Interleukin;* NS, not significant (*p* < 0.05); NA, not applicable; SNP, single nucleotide polymorphism; TLR, Toll-like receptor.

a SNP database (dbSNP) reference number (ID number).

b *p*-value – for comparison between genotypes and viremia.

c *p*-value – for comparison between genotypes and viruria.

**Table S5:** Association between genotypes of examined polymorphisms and viremia and viruriain asymptomaticneonates with cCMV infection

Asymptomatic infants with cCMV infection(*n* = 19)						
SNP, rs number^a^, alleles	Genotype	*n* = 19 *n* (%)	HCMV DNA (copies/mL) in blood	*p-value* ^b^	HCMV DNA (copies/mL) in urine	*p-value* ^c^
IL1B	A/A	2 (10.53)	1.4 × 10^4^ (9.9 × 10^2^–2.7 × 10^4^)		5.8 ×10^6^ (2.8 × 10^6^–8.8 × 10^6^)	
rs16944	A/G	10 (52.63)	1.8 × 10^3^ (3.1 × 10^2^–4.0 × 10^3^)	NS	2.7 ×10^5^ (4.0 × 10^4^–3.2 × 10^6^)	NS
G/A	G/G	7 (36.84)	2.0 × 10^3^ (1.2 × 10^3^–2.1 × 10^3^) (*n* = 5)		6.9 ×10^5^ (1.4 × 10^5^–6.2 × 10^6^)	
IL12B	T/T	14 (73.68)	1.8 × 10^3^ (1.0 × 10^3^–1.4 × 10^4^) (*n* = 12)		4.9 × 10^5^ (1.4 × 10^5^–4.6 × 10^6^)	
rs3212227	T/G	4 (21.05)	2.0 × 10^3^ (0–3.8 × 10^5^)	NS	1.1 × 10^7^ (1.0 × 10^6^–4.0 × 10^7^)	NS
G/T	G/G	1 (5.26)	2.1 × 10^3^ (2.1 × 10^3^–2.1 × 10^3^)		1.7 × 10^5^ (1.7 × 10^5^–1.7 × 10^5^)	
IL28B	T/T	2 (10.53)	1.9 × 10^3^ (1.2 × 10^3^–2.7 × 10^3^)		2.7 × 10^5^ (2.5 × 10^5^–2.9 × 10^5^)	
rs12979860	T/C	6 (31.58)	1.1 × 10^3^ (0–1.6 × 10^3^)	NS	2.4× 10^6^ (1.7 × 10^5^–6.2 × 10^6^)	NS
C/T	C/C	11 (57.89)	2.1 × 10^3^ (2.0 × 10^3^–2.7 × 10^4^) (*n* = 9)		6.9× 10^5^ (4.0 × 10^4^–6.1 × 10^6^)	
CCL2	G/G	0 (0.00)	NA		NA	
rs1024611	G/A	9 (47.37)	2.4 × 10^3^ (9.5 × 10^2^–1.5 × 10^4^) (*n* = 8)	NS	2.5× 10^5^ (1.4 × 10^5^–3.2 × 10^6^)	NS
A/G	A/A	10 (52.63)	1.2 × 10^3^ (9.9 × 10^2^–2.1 × 10^3^) (*n* = 9)		1.7× 10^6^ (1.7 × 10^5^–6.1 × 10^6^)	
DC-SIGN	G/G	7 (36.84)	3.1 × 10^2^ (0–1.2 × 10^3^) (*n* = 5)		2.9× 10^5^ (3.3 × 10^3^–2.0 × 10^7^)	
rs735240	G/A	6 (31.58)	2.0 × 10^3^ (1.6 × 10^3^–2.7 × 10^4^)	NS	2.6× 10^6^ (1.7 × 10^5^–6.2 × 10^6^)	NS
A/G	A/A	6 (31.58)	2.4 × 10^3^ (9.9 × 10^2^–3.2 × 10^4^)		1.1× 10^6^ (4.0 × 10^4^–2.8 × 10^6^)	
TLR2	G/G	17 (89.47)	2.1 × 10^3^ (3.1 × 10^2^–2.7 × 10^4^)(*n* = 15)		2.0× 10^6^ (1.4 × 10^5^–6.1 × 10^6^)	
rs5743708	G/A	2 (10.53)	1.4 × 10^3^ (1.2 × 10^3^–1.6 × 10^3^)	NS	2.3× 10^5^ (1.7 × 10^5^–2.9 × 10^5^)	NS
A/G	A/A	0 (0.00)	NA		NA	
TLR4	T/T	0 (0.00)	NA		NA	
rs4986791	T/C	2 (10.53)	2.0 × 10^3^ (0–4.0 × 10^3^)	NS	1.0 × 10^7^ (0–2.0 × 10^7^)	NS
C/T	C/C	17 (89.47)	2.0 × 10^3^ (9.9 × 10^2^–2.7 × 10^4^)(*n* = 15)		6.9× 10^5^ (1.7 × 10^5^–4.6 × 10^6^)	
TLR9	T/T	7 (36.84)	1.4 × 10^3^ (0–4.0 × 10^3^) (*n* = 6)		2.9× 10^5^ (1.4 × 10^5^–2.0 × 10^7^)	
rs352140	T/C	9 (47.37)	2.1 × 10^3^ (1.6 × 10^3^–1.7 × 10^4^)(*n* = 8)	NS	6.9× 10^5^ (1.7 × 10^5^–4.6 × 10^6^)	NS
C/T	C/C	3 (15.79)	3.1× 10^2^ (0–2.7 × 10^4^)		3.2× 10^6^ (0–8.8 × 10^6^)	

Data are presented as median (IQR- interquartile range) or number (%). cCMV, congenital cytomegalovirus; *CCL2*, C-C *motif* chemokine ligand 2; DC-SIGN, dendritic cell-specific ICAM-grabbing non-integrin; HCMV, human cytomegalovirus; IL*, Interleukin;* NS, not significant (*p* < 0.05); NA, not applicable; SNP, single nucleotide polymorphism; TLR, Toll-like receptor.

a SNP database (dbSNP) reference number (ID number).

b *p*-value – for comparison between genotypes and viremia.

c *p*-value – for comparison between genotypes and viruria.

**Table S6:** Association between alleles of examined polymorphisms (SNPs) and viremia and viruria in newborn infants with cCMV infection

Newborn infants with cCMV infection (*N* = 92)						
SNP, rs number^a^, alleles	Allele	*n*, (%)	HCMV DNA (copies/mL) in blood	*p*-value^b^	HCMV DNA (copies/mL) in urine	*p*-value^c^
IL1B	G	121 (65.76)	1.2 × 10^4^ (2.0 × 10^3^–1.7 × 10^5^) (*n* = 116)		1.0 ×10^7^ (1.1 × 10^6^–1.0 × 10^7^) (*n* = 118)	
rs16944				NS		NS
G/A	A	63 (34.24)	7.6 × 10^3^ (2.4 × 10^3^–3.8 × 10^4^) (*n* = 62)		6.8 ×10^6^ (1.1 × 10^6^–1.0 × 10^7^) (*n* = 62)	
IL12B	T	142 (77.17)	1.6 × 10^4^ (2.0 × 10^3^–1.3 × 10^5^) (*n* = 136)		7.0 ×10^6^ (1.0 × 10^6^–1.0 × 10^7^) (*n* = 139)	
rs3212227				NS		
G/T	G	42 (22.83)	4.8 × 10^3^ (2.1 × 10^3^–3.0 × 10^4^)		1.0 ×10^7^ (1.7 × 10^6^–1.0 × 10^7^) (*n* = 41)	NS
IL28B	C	120 (65.22)	1.1 × 10^4^ (2.1 × 10^3^–9.0 × 10^4^) (*n* = 116)		8.8 ×10^6^ (1.0 × 10^6^–1.0 × 10^7^) (*n* = 119)	
rs12979860				NS		NS
C/T	T	64 (34.78)	8.1 × 10^3^ (1.6 × 10^3^–1.3 × 10^5^) (*n* = 62)		7.0 ×10^6^ (1.3 × 10^6^–1.0 × 10^7^) (*n* = 61)	
CCL2	A	139 (75.54)	1.1 × 10^4^ (2.1 × 10^3^–1.3 × 10^5^) (*n* = 134)		7.0 ×10^6^ (1.1 × 10^6^–1.0 × 10^7^) (*n* = 137)	
rs1024611				NS		NS
A/G	G	45 (24.46)	1.0 × 10^4^ (1.6 × 10^3^–4.9 × 10^4^) (*n* = 44)		9.2 ×10^6^ (8.4 × 10^5^–1.0 × 10^7^) (*n* = 43)	
DC-SIGN	G	107 (58.15)	1.1 × 10^4^ (2.0 × 10^3^–1.3 × 10^5^) (*n* = 101)		9.6 ×10^6^ (1.3 × 10^6^–1.0 × 10^7^) (*n* = 106)	
rs735240				NS		NS
A/G	A	77 (41.85)	9.4 × 10^3^ (2.1 × 10^3^–5.7 × 10^4^)		6.1 ×10^6^ (1.0 × 10^6^–1.0 × 10^7^) (*n* = 74)	
TLR2	G	174 (94.57)	1.1 × 10^4^ (2.1 × 10^3^–1.0 × 10^5^) (*n* = 168)		8.6 ×10^6^ (1.1 × 10^6^–1.0 × 10^7^) (*n* = 170)	
rs5743708				NS		NS
A/G	A	10 (5.43)	4.3 × 10^3^ (1.6 × 10^3^–3.8 × 10^4^)		1.0 ×10^7^ (1.0 × 10^6^–1.0 × 10^7^)	
TLR4	C	175 (95.11)	1.0 × 10^4^ (2.1 × 10^3^–9.8 × 10^4^) (*n* = 169)		8.6 ×10^6^ (1.1 × 10^6^–1.0 × 10^7^) (*n* = 171)	
rs4986791				NS		NS
C/T	T	9 (4.89)	2.0 × 10^4^ (2.8 × 10^3^–9.8 × 10^4^)		1.0 ×10^7^ (1.7 × 10^6^–1.0 × 10^7^)	
TLR9	T	107 (58.15)	1.7 × 10^3^ (2.1 × 10^3^–1.5 × 10^5^) (*n* = 103)		9.2 ×10^6^ (1.1 × 10^6^–1.0 × 10^7^) (*n* = 104)	
rs352140				NS		NS
C/T	C	77 (41.85)	7.9 × 10^3^ (1.7 × 10^3^–5.1 × 10^4^) (*n* = 75)		7.8 ×10^6^ (1.0 × 10^6^–1.0 × 10^7^) (*n* = 76)	

Data are presented as median (IQR- interquartile range) or number (%). cCMV, congenital cytomegalovirus; *CCL2*,C-C *motif* chemokine ligand 2; DC-SIGN, dendritic cell-specific ICAM-grabbing non-integrin; HCMV, human cytomega lovirus; IL*, interleukin;* NS, not significant (*p* < 0.05); SNP, single nucleotide polymorphism;; TLR, toll-like receptor.

a SNP database (dbSNP) reference number (ID number).

b *p*-value – for comparison between alleles and viremia.

c *p*-value – for comparison between alleles and viruria.

**Table S7:** Association between alleles of examined polymorphisms (SNPs) and viremia and viruria in asymptomatic newborn infants with cCMV infection

Asymptomatic newborn infants with cCMV infection (*N* = 19)						
SNP, rs number^a^, alleles	Allele	*n*, (%)	HCMV DNA (copies/mL) in blood	*p*-value^b^	HCMV DNA (copies/mL) in urine	*p*-value^c^
IL1B	G	24 (63.16)	2.0 × 10^3^ (7.5 × 10^2^–3.3 × 10^3^) (*n* = 20)		4.9 ×10^5^ (1.4 × 10^5^–5.3 × 10^6^)	
rs16944				NS		NS
G/A	A	14 (36.84)	1.8 × 10^3^ (9.9 × 10^2^–2.7 × 10^4^)		2.4 ×10^6^ (1.7 × 10^5^–6.1 × 10^6^)	
IL12B	T	32 (84.21)	1.8 × 10^3^ (9.9 × 10^2^–1.5 × 10^4^) (*n* = 28)		6.9 ×10^5^ (1.4 × 10^5^–5.3 × 10^6^)	
rs3212227				NS		NS
G/T	G	6 (15.79)	2.1 × 10^3^ (0.0–4.0 × 10^3^)		1.0 ×10^6^ (1.7 × 10^5^–2.0 × 10^7^)	
IL28B	C	28 (73.68)	2.1 × 10^3^ (6.5 × 10^2^–2.7 × 10^4^) (*n* = 24)		1.3 ×10^6^ (9.1 × 10^4^–6.1 × 10^6^)	
rs12979860				NS		NS
C/T	T	10 (26.32)	1.2 × 10^3^ (9.9 × 10^2^–2.7 × 10^3^)		2.9 ×10^5^ (2.5 × 10^5^–2.8 × 10^6^)	
CCL2	A	29 (76.32)	1.8 × 10^3^ (9.9 × 10^2^–4.0 × 10^3^) (*n* = 26)		6.9 ×10^5^ (1.7 × 10^5^–6.1 × 10^6^)	
rs1024611				NS		NS
A/G	G	9 (23.68)	2.4 × 10^3^ (9.5 × 10^2^–1.5 × 10^4^) (*n* = 8)		2.5 ×10^5^ (1.4 × 10^5^–3.2 × 10^6^)	
DC-SIGN	G	20 (52.63)	1.2 × 10^3^ (1.5 × 10^2^–3.0 × 10^3^) (*n* = 16)		4.9 ×10^5^ (1.4 × 10^5^–7.5 × 10^6^)	
rs735240				NS		NS
A/G	A	18 (47.37)	2.1 × 10^3^ (1.2 × 10^3^–3.2 × 10^4^)		1.3 ×10^6^ (1.7 × 10^5^–4.6 × 10^6^)	
TLR2	G	36 (94.74)	2.0 × 10^3^ (6.5 × 10^2^–1.5 × 10^4^) (*n* = 32)		1.3 ×10^6^ (1.4 × 10^5^–6.1 × 10^6^)	
rs5743708				NS		NS
A/G	A	2 (5.26)	1.4 × 10^3^ (1.2 × 10^3^–1.6 × 10^3^)		2.3 ×10^5^ (1.7 × 10^5^–2.9 × 10^5^)	
TLR4	C	36 (94.74)	2.0 × 10^3^ (9.9 × 10^2^–1.5 × 10^4^) (*n* = 32)		6.9 ×10^5^ (1.5 × 10^5^–5.3 × 10^6^)	
rs4986791				NS		NS
C/T	T	2 (5.26)	2.0 × 10^3^ (0–4.0 × 10^3^)		1.0 ×10^7^ (0–2.0 × 10^7^)	
TLR9	T	23 (60.53)	1.8 × 10^3^ (1.0 × 10^3^–4.0 × 10^3^) (*n* = 20)		2.9 ×10^5^ (1.4 × 10^5^–6.1 × 10^6^)	
rs352140				NS		NS
C/T	C	15 (39.47)	2.0 × 10^3^ (3.1 × 10^2^–2.7 × 10^4^) (*n* = 14)		2.8 ×10^6^ (4.0 × 10^4^–6.1 × 10^6^)	

Data are presented as median (IQR- interquartile range) or number (%). cCMV, congenital cytomegalovirus; *CCL2*, C-C *motif* chemokine ligand 2; DC-SIGN, dendritic cell-specific ICAM-grabbing non-integrin; HCMV, human cytomega lovirus; IL*, interleukin;* NS, not significant (*p* < 0.05); SNP, single nucleotide polymorphism; TLR, toll-like receptor.

a SNP database (dbSNP) reference number (ID number).

b *p*-value – for comparison between alleles and viremia.

c *p*-value – for comparison between alleles and viruria.
